# Human profiling from STR and SNP analysis of tropical bed bug, *Cimex hemipterus*, for forensic science

**DOI:** 10.1038/s41598-023-28774-y

**Published:** 2023-01-27

**Authors:** Li Lim, Abdul Hafiz Ab Majid

**Affiliations:** 1 Household and Structural Urban Entomology Laboratory, Vector Control Research Unit, School of Biological Sciences, Universiti Sains Malaysia, 11800 Penang, Minden, Malaysia; 2 Centre for Insect Systematics, Faculty of Science and Technology, Universiti Kebangsaan Malaysia (UKM), Selangor, Malaysia

**Keywords:** Biotechnology, Genetics

## Abstract

Tropical bed bugs, *Cimex hemipterus*, which commonly feeds on human blood, may be useful in forensic applications. However, unlike the common bed bug, *Cimex lectularius*, there is no information regarding tropical bed bug, *C. hemipterus*, being studied for its applications in forensics. Thus, in this study, lab-reared post-feeding tropical bed bugs were subjected to Short Tandem Repeat (STR) and Single Nucleotide Polymorphism (SNP) analyses to establish the usage of tropical bed bugs in forensics. Several post-feeding times (0, 5, 14, 30, and 45 days) were tested to determine when a complete human DNA profile could still be obtained after the bugs had taken the blood meal. The results showed that complete STR and SNP profiles could only be obtained from the D0 sample. The profile completeness decreased over time, and partial STR and SNP profiles could be obtained up to 45 days post-blood meal. The generated SNP profiles, complete or partial, were also viable for HIrisPlex-S phenotype prediction. In addition, field-collected bed bugs were also used to examine the viability of the tested STR markers, and the STR markers detected mixed profiles. The findings of this study established that the post-blood meal of tropical bed bugs is a suitable source of human DNA for forensic STR and SNP profiling. Human DNA recovered from bed bugs can be used to identify spatial and temporal relations of events.

## Introduction

Biological fluids such as blood, semen, or body tissues found at a crime scene are sources for DNA samples. However, there are cases where no corpses or noticeable biological stains could be found at the crime scenes^1,2^. Other sources found on the site, like insects which commonly consume human biological fluid, can be considered in such cases^3^. Using DNA typing, the human DNA retrieved from these insects can be used to identify the individuals on whom these insects have been feeding. Therefore, these sources could serve as invaluable evidence in criminal investigations, as well as helpful in identifying missing corpses that have been removed from a crime scene by providing useful information, particularly on whether the insects have been feeding on multiple food sources^4,5^.

As obligate hematophagous insects that commonly feed on human blood, bed bugs have the potential to be used in forensic studies. Unlike other blood-feeding insects such as mosquitoes which only females feed on blood, all life stages of bed bugs, including all stages of nymphs and adults, are fed on blood to grow, survive, and reproduce^6^. They also tend to aggregate in harborages after blood-feeding. Moreover, bed bugs have a relatively long lifespan and survive longer without a blood meal compared to mosquitoes, fleas, and lice. Thus, there is a greater chance of finding recently fed bed bugs on the site^7,8^. In addition, bed bugs are wingless insects that are most likely to remain close to their feeding location^8^. Other than this, the possibility of bed bugs appearing in a particular crime scene could be high, with the recent resurgence of infestation of bed bugs.

Nowadays, Short Tandem Repeats (STR) are the most analyzed genetic polymorphism in forensic genetics because they are highly discriminatory^9^. Besides STR analysis, Single Nucleotide Polymorphism (SNP) analysis is an alternative way for identity profiling. It is always used when positive results cannot be obtained from the STR analysis. Some SNP markers can be used in an individual's biogeographical ancestry and phenotypes, such as hair, eye, and skin colour predictions, provide additional investigation leads.

The objective of this study is to determine whether both STR and SNP profiles can be generated from human DNA samples acquired from tropical bed bugs, *Cimex hemipterus*, which is one of the most common bed bugs species found in Malaysia. In order to determine the temporal effects on amplification success of the STR and SNP markers, laboratory strain tropical bed bugs were used, and the DNA typing was done on 0, 5, 14, 30, and 45 days after the bed bugs’ blood-feeding process. Thirteen CODIS (Combined DNA Index System) loci, namely, D18S51, D8S1117, vWA, D21S11, TH01, CSF1PO, D13S317, D7S820, D5S818, D3S1358, FGA, TPOX, D16S539, and one Y chromosome locus, DYS393 were used for the STR profiling, while 41 phenotype-related SNP were used for the SNP profiling. The same STR markers were also applied to the field-collected tropical bed bugs to see if they worked and, if so, what the results were.

## Materials and methods

### Tropical bed bugs, *Cimex hemipterus*

The tropical bed bugs used were a laboratory strain maintained at the Household and Structural Urban Entomology Laboratory, School of Biological Sciences, Universiti Sains Malaysia^10^. The bed bug colony was established from the specimens collected initially in 2014 from cushioned seats in the waiting area located at the Kuala Lumpur International Airport (KLIA), Malaysia, in which they were labeled as the KLIA strain^11^. The bed bugs were reared in 200 mℓ plastic containers stored in an incubator at 23 ± 1 °C. They were released to feed on a human volunteer's arm once a week. Informed consent was obtained from the volunteer, and the feeding methods were carried out following relevant guidelines and regulations. The Human Ethics Committee of Universiti Sains Malaysia (USM/JEPeM/19120868) also approved the experimental protocols.

One bed bug was placed in a 1.5 mℓ microcentrifuge tube, preserved in absolute ethanol with three replications; 0, 5, 14, 30, and 45 days after blood feeding; and then stored at − 20 °C until the DNA extraction.

Other tropical bed bugs were also collected from the field in six areas of Penang, Malaysia, where bed bugs infestation had been reported (Table S1). All bed bug strains were euthanized with absolute ethanol and preserved in a − 20 °C freezer. Three samples were randomly selected from each location to proceed with DNA extraction, followed by STR profiling.

### DNA extraction

The absolute ethanol used to preserve the bed bugs was first removed. Next, sterilized distilled water was added into the tube, which was vortexed to remove any traces of ethanol. The cleaning process was repeated twice. Then, the bed bugs were crushed, and the DNA was extracted using the GF-1 Blood DNA extraction kit (Vivantis Technology, USA), in accordance with the manufacturer’s protocol.

### STR loci

The 13 STR markers and one Y chromosome marker used in this study are listed in Table S2. The sets and sequences of primers were acquired from the STRBase database. All the samples were genotyped in a singleplex reaction, and a Thermal Cycler (TaKaRa, Japan) was used for the PCR amplification. The cycle of PCR was set at 94 °C for denaturing (30 s) with varied annealing temperature of one min (Table S2), and 72 °C for elongation (5 min). The PCR reactions with no DNA sample were used as the negative control.

Next, the PCR products were subjected to the capillary electrophoresis on a Fragment Analyzer (Agilent, US). The observed peaks were then cross-referenced with the STRBase to obtain the allelic number.

### SNP loci

In this study, 41 SNPs were tested by following the HIrisPlex-S system^12–14^. The primer pairs for each SNP are listed in Table S3. The primer sequences were obtained from the NCBI database or designed using Primer3^15^. All the samples were genotyped in a singleplex reaction. Thermal Cycler (TaKaRa, Japan) was used for the PCR amplification, and the cycle of PCR was set at 94 °C for denaturing (30 s) with varied annealing temperature of one min (Table S3) and 72 °C for elongation (5 min). The PCR reactions with no DNA sample were used as the negative control. The PCR products were then sent to Apical Scientific SDN. BHD. (Selangor, Malaysia) for sequencing.

### STR and SNP profile completeness

The number of successfully amplified loci from each sample was recorded. Next, the observed peaks were cross-checked with the STRBase database to obtain the allelic number. For lab-reared samples, symbols were used to represent the numbers of readable alleles (Table 1). For instance, (++) represent both alleles of heterozygous or homozygous loci were readable, (+/−) indicate only one allele of heterozygous or homozygous loci was readable, and (−/−) showed both alleles were not detectable. The completeness of the STR profiles was then presented in percentage values by performing the following calculation^16^:

$${\text{Profile}}\;{\text{completeness}}\;\left( \% \right) = \left( {{\text{Number}}\;{\text{of}}\;{\text{loci}}\;{\text{that}}\;{\text{amplified}}} \right)/\left( {{\text{Total}}\;{\text{number}}\;{\text{of}}\;{\text{loci}}} \right) \times {1}00$$

**Table 1:** Human STR analysis of blood cellular DNA drawn from the tropical bed bugs.

STR locus	Time after blood meal ingestion (days)				
	0 (D0)	5 (D5)	14 (D14)	30 (D30)	45 (D45)
D18S51	++	++	++	++	− / −
D8S1117	++	++	++	++	++
vWA	++	++	++	++	++
D21S11	++	++	− / −	− / −	++
TH01	++	++	++	++	++
CSF1PO	++	− / −	− / −	− / −	− / −
D13S317	++	++	++	++	++
D7S820	++	++	++	− / −	− / −
D5S818	++	− / −	− / −	− / −	− / −
D3S1358	++	++	− / −	− / −	− / −
FGA	++	− / −	− / −	− / −	− / −
TPOX	++	− / −	− / −	++	++
D16S539	++	++	++	++	++
DYS393	++	++	++	++	++
**% (14/14)**	**100.00**	**71.43**	**57.14**	**57.14**	**57.14**

(++) both alleles of heterozygous or homozygous loci were readable.

(+/−) only one allele of heterozygous or homozygous loci was readable.

(−/−) both alleles were not detectable.

(%) percentage of profile completeness.

Significant values are in bold

As for field collected samples, the numbers of readable alleles were presented (Table 2).

**Table 2:** Allelic data for the field-collected samples.

No. of alleles	L1			L2			L3			L4			L5			L6		
	S1	S2	S3	S1	S2	S3	S1	S2	S3	S1	S2	S3	S1	S2	S3	S1	S2	S3
D18S51	–	3	3	1	4	4	3	4	3	2	1	3	–	4	3	1	1	–
D8S1179	2	1	1	4	1	1	3	2	1	3	–	–	2	2	5	3	–	–
vWA	2	6	1	5	–	2	5	2	3	3	4	1	3	6	4	3	–	3
D21S11	–	2	3	2	4	1	4	3	4	–	1	2	1	3	–	–	1	2
TH01	1	–	2	1	2	2	4	1	2	3	2	2	2	2	1	3	2	–
CSF1PO	1	2	2	2	2	2	2	2	1	1	–	–	2	1	1	–	–	–
D13S317	1	2	2	3	2	2	5	2	2	1	3	2	1	2	2	1	2	4
D7S820	–	2	1	–	1	1	–	1	1	–	1	3	–	2	1	–	–	–
D5S818	–	4	1	2	4	4	3	3	3	1	3	2	1	3	2	1	1	1
D3S1358	5	2	–	2	4	1	4	2	–	3	1	–	3	3	–	3	2	–
FGA	1	–	4	5	4	5	4	6	7	6	1	6	7	6	3	7	3	1
TPOX	–	3	3	–	3	2	1	2	2	–	2	–	–	2	4	1	2	3
D16S539	6	1	3	5	1	3	3	3	3	3	2	4	4	3	3	4	5	1
DY	–	–	3	–	1	–	–	1	3	1	–	2	2	3	–	–	–	–

“S” refers to “sample”; “–” indicated no result.

### SNP profile on phenotype prediction

The generated SNP profile was uploaded to HIrisPlex-S Eye, Hair and Skin Colour DNA Phenotyping Webtool^12,14^ to predict the eye, hair, and skin color of the DNA sample.

## Results

### Temporal effects on the STR profile completeness

The results in Table 1 showed that only the samples on Day 0 (D0) generated a complete profile (all tested loci showed peak/s, and each peak could be assigned with an allelic number from the STRBase database). Meanwhile, partial profiles were obtained from the samples on day 5 (D5), 14 (D14), 30 (D30), and 45 (D45), with the number of loci with readable alleles decreasing. Thus, it can be inferred from these findings that the percentage of profile completeness decreased over time. For example, for D5 samples, the peaks at CSF1PO, D5S818, FGA, and TPOX were missed (partial profile). None of the loci were amplified in the negative control samples.

### Allelic data on the field-collected tropical bed bugs

Allelic data on the field-collected tropical bed bugs are presented in Table 2. The numbers represent the number of alleles observed at each locus. For example, the number “2” for L1S1 at the vWA locus indicates that two alleles were detected. More than two alleles could be observed in some loci, suggesting that the bed bugs may have fed on more than two people whose DNA was identified. Dashes in the table indicate that no alleles were observed for the respective locus.

### Temporal effect on SNP profile completeness

Table 3 indicates that all SNP on D0 could be detected, while for D5 and D14 samples, only SNP, rs1126809, were not available. The number of undetected SNP increased for the samples on D30 (rs1126809 and rs3212355) and D45 (rs4959270, rs2402130, rs12913832, rs1393350, and rs1126809).

**Table 3:** Human SNP analysis of blood cellular DNA drawn from the tropical bed bugs.

No.	Locus	SNP	Sample/allele				
			D0	D5	D14	D30	D45
1	MC1R	rs312262906	C	C	C	C	C
2	MC1R	rs11547464	C	C	C	C	C
3	MC1R	rs885479	C	C	C	C	C
4	MC1R	rs1805008	C	C	C	C	C
5	MC1R	rs1805005	A	A	A	A	A
6	MC1R	rs1805006	T	C	C	C	C
7	MC1R	rs1805007	C	C	C	C	C
8	TUBB3	rs1805009	G	G	G	G	G
9	MC1R	rs201326893	C	C	C	C	C
10	MC1R	rs2228479	A	A	A	A	A
11	MC1R	rs1110400	T	T	T	T	T
12	SLC45A2	rs28777	C	C	C	C	C
13	SLC45A2	rs16891982	C	C	C	C	C
14	KITLG	rs12821256	T	T	T	T	T
15	LOC105374875	rs4959270	C	C	C	C	–
16	IRF4	rs12203592	C	C	C	C	C
17	TYR	rs1042602	C	C	C	C	C
18	OCA2	rs1800407	T	T	T	T	T
19	SLC24A4	rs2402130	A	A	A	A	–
20	HERC2	rs12913832	A	A	A	A	–
21	PIGU	rs2378249	A	A	A	A	A
22	LOC105370627	rs12896399	G	G	G	G	G
23	TYR	rs1393350	G	G	G	G	–
24	TYRP1	rs683	C	C	C	C	C
25	ANKRD11	rs3114908	C	C	C	C	C
26	OCA2	rs1800414	C	C	C	C	C
27	BNC2	rs10756819	A	A	A	A	A
28	HERC2	rs2238289	G	G	G	G	G
29	SLC24A4	rs17128291	T	T	T	T	T
30	HERC2	rs6497292	G	G	G	G	G
31	HERC2	rs1129038	G	G	G	G	G
32	HERC2	rs1667394	T	T	T	T	T
33	TYR	rs1126809	G	–	–	–	–
34	OCA2	rs1470608	T	T	T	T	T
35	SLC24A5	rs1426654	A	A	A	A	A
36	ASIP	rs6119471	C	C	C	C	C
37	OCA2	rs1545397	T	T	T	T	T
38	RALY	rs6059655	G	G	G	G	A
39	OCA2	rs12441727	G	G	G	G	G
40	MC1R	rs3212355	C	C	C	-	C
41	DEF8	rs8051733	A	A	A	A	A
**% (41/41)**			**100.00**	**97.56**	**97.56**	**95.12**	**87.80**

Significant values are in bold

### Phenotype prediction

Next, the obtained SNP profile presented in Table 3 was entered into the HIrisPlex-S System, a web tool for eye, hair, and skin color DNA phenotyping. Based on the DNA phenotyping results presented in Table 4, the most likely predicted phenotype for D0 samples matched the appearance characteristics of the volunteer on whom the bed bug samples had fed. Although there was AUC loss with two SNPs missing in the D5 and D14 samples, the outcomes of the phenotype prediction were similar to those observed in the D0 samples. For the D30 samples, although there was also AUC loss due to missing two SNP, the phenotype prediction was still accurate. However, for the D45 samples, the eye color prediction could not be produced. Furthermore, with a total of five missing SNP, the hair and skin color were far from the prediction as the D0 samples, despite the AUC loss was acceptable (Table 4).

**Table 4:** Eye, hair, and skin color prediction results from the laboratory bed bug samples at different post-feeding time interval.

Phenotype/Category		Sample									
		D0		D5		D14		D30		D45	
		*p* value	AUC loss	*p* value	AUC loss	*p* value	AUC loss	*p* value	AUC loss	*p* value	AUC loss
Eye color	Blue eye	0.338	0	0.338	0	0.338	0	0.338	0	0	0.296
	Intermediate eye	0.115	0	0.115	0	0.115	0	0.115	0	0	0.139
	Brown eye	0.547	0	0.547	0	0.547	0	0.547	0	0	0.299
Hair color	Blond hair	0.133	0	0.124	0	0.124	0	0.124	0	0.023	0.061
	Brown hair	0.529	0	0.43	0	0.43	0	0.43	0	0.236	0.046
	Red hair	0.003	0	0.005	0	0.005	0	0.005	0	0.001	0.003
	Black hair	0.336	0	0.442	0	0.442	0	0.442	0	0.741	0.045
Shade	Light hair	0.322	0	0.295	0	0.295	0	0.295	0	0.065	0.076
	Dark hair	0.678	0	0.705	0	0.705	0	0.705	0	0.935	0.076
Skin color	Very pale skin	0.145	0	0.389	0.01	0.389	0.01	0.411	0.011	0.687	0.018
	Pale skin	0.007	0	0.008	0.002	0.008	0.002	0.022	0.002	0.019	0.004
	Intermediate skin	0.846	0	0.603	0.001	0.603	0.001	0.566	0.001	0.293	0.005
	Dark skin	0.002	0	0	0	0	0	0	0.001	0	0
	Dark to black skin	0	0	0	0	0	0	0	0	0	0.002
Most likely predicted phenotype		Brown eye, dark brown/black hair, pale skin		Brown eye, dark brown/black hair, pale skin		Brown eye, dark brown/black hair, pale skin		Brown eye, dark brown/black hair, pale skin		No eye colors, black/dark brown hair, pale skin	

The eye color prediction results consisted of three categories, including blue, intermediate (non-blue and non-brown), and brown, with a *p* value that sums up to 1. The hair color prediction result consisted of four categories: blond, brown, red, and black, with a *p* value close to 1. There was also a shade prediction probability (*p* value) for light and dark that summed up to 1. The skin color prediction result consisted of five categories: very pale, pale, intermediate, dark, and dark-to-black, with a total *p* value of 1.

The prediction accuracy expressed as the area under the receiver operating characteristics curve (AUC) of 0.5 represents a random prediction, and 1.0 represents a completely accurate prediction. The AUC loss for each prediction represents the loss in AUC accuracy when using all the required SNPs.

## Discussion

This study was the first to focus on acquiring human DNA from tropical bed bugs, *C. hemipterus* for STR and SNP profiling. The results showed that DNA recovered from the tropical bed bugs could be amplified, analyzed, and used for individual identification. Partial profiles of both STR and SNP could be generated up to 45 days after the tropical bed bugs' blood feeding. The outcome of this study was promising compared to results acquired from other insects, such as mosquito blood, where the human DNA could only be detected up to 26 h after ingestion^17^. However, the limitation of this study is using singleplex PCR for DNA profiling instead of the commercial multiplex kits. As the protocol used was different, the amplification efficiency could be the same or different from the multiplex kits.

Except for D0 samples, only incomplete STR profiles were observed in this study, with the amplified loci decreasing over time. This is in accordance with the studies by Schal^18^ and Njau^19^ on common bed bugs and maggots, in which the recovered amount of DNA was found to be declining over time. The DNA typing failure may be due to the low amount of DNA obtained after extraction^4^. Another possible explanation is the high DNA degradation within the tropical bed bugs caused by gut enzymes or nucleases^4,19^. Therefore, crime scene investigators should be able to distinguish the larger and bright red engorged bed bugs from the unengorged ones, and to try their best to collect the former ones in order to increase the chances of human DNA isolation and DNA typing^7^.

According to Machida and Kibayashi^20^, STR with shorter amplicon sizes was easier to amplify. Based on Table 1, large loci, including FGA (average: 240 bp), D7S820 (average: 276 bp), D18S51 (average: 295 bp), and CSF1PO (average: 325 bp), did follow the trend which was not observed in supposed more severe DNA samples. However, the loci amplification should be more fortuitous; for example, while peaks for locus D21S11 were not observed on the D14 and D30 samples, they were observed on the D45 samples. Furthermore, for locus TPOX, no peak was observed on the D5 and D14 samples, but the peaks were amplified on the D30 and D45 samples.

The mixed profile could be observed from the field-collected samples as there were more than two peaks or alleles for some of the tested loci. Repeated analysis is conducted to eliminate the possibility that these additional bands could be stutter peaks^21^. This showed that the STR markers could be used to identify more than one host from a single bed bug. Although bed bugs are most likely to complete a blood meal in a single feeding bout, they can sometimes be disrupted during the feeding, and then resume the feeding on a different host^18^, resulting in mixed profiles, as observed in this study. Alternatively, it could also be that the DNA belongs to previous different hosts before their most recent meal. This suggests that more research is needed to confirm whether human DNA from their previous feeding can still be found after the next meal.

The possibility of posing mixed DNA within the tropical bed bug may hinder the application of this method in actual cases. Nevertheless, mixed DNA samples are common in forensic casework samples, and using tropical bed bugs as an alternative human DNA storage for STR typing could benefit in certain conditions. This could also expand the scope of human STR profiling to common blood-feeding urban pests.

Similar to STR profiling, the profile completeness of SNP profiling also decreased over time, although the SNP typing should be more resistant to DNA degradation due to their shorter amplicons^22^. The HIrisPlex-S phenotype prediction for D0 samples with complete SNP profile matched the skin, eyes, and hair colors of the volunteer. Most phenotype predictions were still accurate for the partial profiles of the D5, D14, and D30 samples, even though with some AUC loss. There was no eye color prediction for D45 sample due to the missing HERC2 rs12913832 which plays a significant role in the functional aspects of iris pigmentation^23^. Thus, this online tool can produce a conservative prediction with partial profiles as long as the critical SNP is present.

The homozygous genotype of SNP, rs28777, and rs16891982 contributes to the hair color prediction as dark brown or black, which was consistent with the volunteer's appearance^24^. For skin color, although all samples (except D45 samples) had the highest *p* value for "intermediate skin", the most likely predicted skin color would be "pale skin," since the predicted color would be affected by the second-highest category (which is the “very pale skin” in this study) if the *p* value is > 0.15, making the skin appear darker^14^. Since STR profiling on the field samples has revealed a mixture of DNA and these SNP phenotyping markers could not classify the admixed samples, the HIrisPlex-S phenotype prediction was not conducted on the field samples.

## Conclusion

Results in this study revealed that human DNA can be acquired from the tropical bed bugs, *C. hemipterus*, and the obtained DNA is sufficient for both STR and SNP profiling. Although the profile completeness decreased over time, partial STR and SNP profiles could still be generated up to 45 days from the tropical bed bugs post-blood meal to be used for personal identification as well as phenotype prediction. Therefore, it can be concluded that bed bugs found in the surrounding area of a crime scene can potentially be used for human DNA profiling in order to obtain evidence or leads in furthering the investigations.

## Supplementary Information

Supplementary Tables.

## Data Availability

The datasets generated and/or analysed during the current study are available in the figshare repository, https://figshare.com/articles/dataset/STR_profile_interpretation_of_DNA_samples_from_field-collected_tropical_bed_bugs/20109800.
