# Supplementary material for: Human profiling from STR and SNP analysis of tropical bed bug, Cimex hemipterus, for forensic science
Source: Sci Rep. 2023 Jan 27;13:1506. doi: 10.1038/s41598-023-28774-y (PMC9883228; doi:10.1038/s41598-023-28774-y)
Supplement: Supplementary file 1 — Supplementary Tables. [file 41598_2023_28774_MOESM1_ESM.docx]

Table S1. Tropical bed bugs collected from six locations in Penang, Malaysia.

| Code | Location | Building type/Premises | Coordinate | Number of samples |
| --- | --- | --- | --- | --- |
| L1 | Jelutong | Shop lot | 5°22'18.7"N 100°18'26.9"E | 8 |
| L2 | Gat Lebuh Macallum | Apartment | 5°24'32.3"N 100°20'03.5"E | 5 |
| L3 | Bukit Mertajam | Shopping mall | 5°22'08.0"N 100°24'53.5"E | 3 |
| L4 | Gelugor | Flat | 5°20'18.5"N 100°18'08.8"E | 4 |
| L5 | Gelugor | Terrace house | 5°22'16.5"N 100°18'28.4"E | 6 |
| L6 | Bayan Lepas | Condominium | 5°19'35.6"N 100°16'48.8"E | 5 |

Table S2. List of STR markers used as retrieved from the STRBase.

| STR locus | Allele range | Primer/Sequence | Reference | ℃ |
| --- | --- | --- | --- | --- |
| D18S51 | 7 to 39.2 | CAAACCCGACTACCAGCAAC | ^10^ | 55 |
|  |  | GAGCCATGTTCATGCCACTG |  |  |
| D8S1179 | 7 to 20 | TTTTTGTATTTCATGTGTACATTCG | ^10^ | 43 |
|  |  | CGTAGCTATAATTAGTTCATTTTCA |  |  |
| vWA | 10 to 25 | CCCTAGTGGATAAGAATAATC | ^11^ | 45 |
|  |  | GGACAGATGATAAATACATAGGATGGATGG |  |  |
| D21S11 | 12 to 41.2 | GTGAGTCAATTCCCCAAG | ^12^ | 45 |
|  |  | GTTGTATTAGTCAATGTTCTCC |  |  |
| TH01 | 3 to 14 | GTGGGCTGAAAAGCTCCCGATTAT | ^13^ | 57 |
|  |  | ATTCAAAGGGTATCTGGGCTCTGG |  |  |
| CSF1PO | 5 to 16 | AACCTGAGTCTGCCAAGGACTAGC | ^14^ | 58 |
|  |  | TTCCACACACCACTGGCCATCTTC |  |  |
| D13S317 | 5 to 17 | ACAGAAGTCTGGGATGTGGA | ^15^ | 49 |
|  |  | GCCCAAAAAGACAGACAGAA |  |  |
| D7S820 | 5 to 16 | TGTCATAGTTTAGAACGAACTAACG | ^15^ | 48 |
|  |  | CTGAGGTATCAAAAACTCAGAGG |  |  |
| D5S818 | 6 to 18 | GGGTGATTTTCCTCTTTGGT | ^15^ | 48 |
|  |  | TGATTCCAATCATAGCCACA |  |  |
| D3S1358 | 8 to 20 | ACTGCAGTCCAATCTGGGT | ^16^ | 46 |
|  |  | ATGAAATCAACAGAGGCTTG |  |  |
| FGA | 12.2 to 51.2 | GCCCCATAGGTTTTGAACTCA | ^17^ | 50 |
|  |  | TGATTTGTCTGTAATTGCCAGC |  |  |
| TPOX | 4 to 16 | CACTAGCACCCAGAACCGTC | ^18^ | 52 |
|  |  | CCTTGTCAGCGTTTATTTGCC |  |  |
| D16S539 | 4 to 16 | GATCCCAAGCTCTTCCTCTT | - | 52 |
|  |  | ACGTTTGTGTGTGCATCTGT |  |  |
| DYS393 | 9 to 17 | GTGGTCTTCTACTTGTGTCAATAC | ^19^ | 44 |
|  |  | AACTCAAGTCCAAAAAATGAGG |  |  |

℃ – Annealing temperature during PCR.

Table S3. List of SNP loci.

| No. | Loci | SNP | Primers/Sequence | | ℃ |
| --- | --- | --- | --- | --- | --- |
| 1. | MC1R | rs312262906 | Forward | CTCAACTCCACCCCCACAG | 60 |
|  |  |  | Reverse | AGGAAGAGCCCGTCAGAGAT |  |
| 2 | MC1R | rs11547464 | Forward | CTGTCCAGCCTCTGCTTCCT | 63 |
|  |  |  | Reverse | GGCCACGTGGTCGTAGTAGG |  |
| 3 | MC1R | rs885479 | Forward | TTCTACGCACTGCGCTACC | 60 |
|  |  |  | Reverse | CGTGCTGAAGACGACACTGG |  |
| 4 | MC1R | rs1805008 | Forward | CTGTCCAGCCTCTGCTTCCT | 63 |
|  |  |  | Reverse | GGCCACGTGGTCGTAGTAGG |  |
| 5 | MC1R | rs1805005 | Forward | CTGGTGAGCTTGGTGGAGA | 60 |
|  |  |  | Reverse | TCCAGCAGGAGGATGACG |  |
| 6 | MC1R | rs1805006 | Forward | CTGGTGAGCTTGGTGGAGA | 60 |
|  |  |  | Reverse | TCCAGCAGGAGGATGACG |  |
| 7 | MC1R | rs1805007 | Forward | CTGTCCAGCCTCTGCTTCCT | 63 |
|  |  |  | Reverse | GGCCACGTGGTCGTAGTAGG |  |
| 8 | TUBB3 | rs1805009 | Forward | GAACTTCAACCTCTTTCTCGCC | 58 |
|  |  |  | Reverse | CCTGGCTGTGGAAGGCGTA |  |
| 9 | MC1R | rs201326893 | Forward | TGGACCGCTACATCTCCATCTT | 59 |
|  |  |  | Reverse | GCCGCAACGGCTCGC |  |
| 10 | MC1R | rs2228479 | Forward | CTGTCCAGCCTCTGCTTCCT | 63 |
|  |  |  | Reverse | GGCCACGTGGTCGTAGTAGG |  |
| 11 | MC1R | rs1110400 | Forward | CTGTCCAGCCTCTGCTTCCT | 63 |
|  |  |  | Reverse | GGCCACGTGGTCGTAGTAGG |  |
| 12 | SLC45A2 | rs28777 | Forward | TACTCGTGTGGGAGTTCCAT | 54 |
|  |  |  | Reverse | TCTTTGATGTCCCCTTCGAT |  |
| 13 | SLC45A2 | rs16891982 | Forward | ATCAAATCCAAGTTGTGCTAGACC | 57 |
|  |  |  | Reverse | CTATAGTGCACACAACTCCACAGAG |  |
| 14 | KITLG | rs12821256 | Forward | ATGCCCAAAGGATAAGGAAT | 52 |
|  |  |  | Reverse | GGAGCCAAGGGCATGTTACT |  |
| 15 | LOC105374875 | rs4959270 | Forward | TGAGAAATCTACCCCCACGA | 58 |
|  |  |  | Reverse | GTGTTCTTACCCCCTGTGGA |  |
| 16 | IRF4 | rs12203592 | Forward | AGGGCAGCTGATCTCTTCAG | 57 |
|  |  |  | Reverse | GCTTCGTCATATGGCTAAACCT |  |
| 17 | TYR | rs1042602 | Forward | CAACACCCATGTTTAACGACA | 51 |
|  |  |  | Reverse | GCTTCATGGGCAAAATCAAT |  |
| 18 | OCA2 | rs1800407 | Forward | TAGTTTGGCTCCCTGTTCTTAAAGT | 57 |
|  |  |  | Reverse | CGATGAGACAGAGCATGATGA |  |
| 19 | SLC24A4 | rs2402130 | Forward | ACCTGTCTCACAGTGCTGCT | 56 |
|  |  |  | Reverse | TTCACCTCGATGACGATGAT |  |
| 20 | HERC2 | rs12913832 | Forward | TCAACATCAGGGTAAAAATCATGT | 50 |
|  |  |  | Reverse | GGCCCCTGATGATGATAGC |  |
| 21 | PIGU | rs2378249 | Forward | CGCATAACCCATCCCTCTAA | 55 |
|  |  |  | Reverse | CATTGCTTTTCAGCCCACAC |  |
| 22 | LOC105370627 | rs12896399 | Forward | CTGGCGATCCAATTCTTTGT | 55 |
|  |  |  | Reverse | GACCCTGTGTGAGACCCAGT |  |
| 23 | TYR | rs1393350 | Forward | TTTCTTTATCCCCCTGATGC | 52 |
|  |  |  | Reverse | GGGAAGGTGAATGATAACACG |  |
| 24 | TYRP1 | rs683 | Forward | CACAAAACCACCTGGTTGAA | 54 |
|  |  |  | Reverse | TGAAAGGGTCTTCCCAGCTT |  |
| 25 | ANKRD11 | rs3114908 | Forward | CAGAACACAGCCACACCCTA | 58 |
|  |  |  | Reverse | CATAAAGGGGTCACCAGCAA |  |
| 26 | OCA2 | rs1800414 | Forward | GCTGCAGGAGTCAGAAGGTT | 55 |
|  |  |  | Reverse | GGGACAAACGAATTGAGGAA |  |
| 27 | BNC2 | rs10756819 | Forward | AAAGCAAGCTCATGTTTCCA | 54 |
|  |  |  | Reverse | CGTCATGACTAGAAAAACACCAA |  |
| 28 | HERC2 | rs2238289 | Forward | GGAGTATGTGGGTTTGTTTCTTAATC | 55 |
|  |  |  | Reverse | GGTCTGCTGTCACTGCTCATT |  |
| 29 | SLC24A4 | rs17128291 | Forward | CCAGCACTGCCAAAATAACA | 55 |
|  |  |  | Reverse | CTCTTTGGACCCATCACCTC |  |
| 30 | HERC2 | rs6497292 | Forward | TCTGCTGTAGAACCAATGTCC | 57 |
|  |  |  | Reverse | GAATTGCACCTGTAGCTCCAT |  |
| 31 | HERC2 | rs1129038 | Forward | ATGTCGACTCCTTTGCTTCG | 58 |
|  |  |  | Reverse | ACACCAGGCAGCCTACAGTC |  |
| 32 | HERC2 | rs1667394 | Forward | CAGCTGTAGAGAGAGACTTTGAGG | 56 |
|  |  |  | Reverse | CACCATTAAGACGCAGCAAT |  |
| 33 | TYR | rs1126809 | Forward | TGTTTCTTAGTCTGAATAACCTTTTCC | 53 |
|  |  |  | Reverse | GGTGCATTGGCTTCTGGATA |  |
| 34 | OCA2 | rs1470608 | Forward | TTTCTTGTGTTAACTGTCCTTACAAA | 53 |
|  |  |  | Reverse | GGAAAATATGTTAGGGTTGATGG |  |
| 35 | SLC24A5 | rs1426654 | Forward | TTCAGCCCTTGGATTGTCTC | 56 |
|  |  |  | Reverse | TGAGTAAGCAAGAAGTATAAGGAGCA |  |
| 36 | ASIP | rs6119471 | Forward | GCAGGAGAATTGCTGGAACT | 55 |
|  |  |  | Reverse | AACCCGAAGGAAGAGTGAAAA |  |
| 37 | OCA2 | rs1545397 | Forward | GGTATAGGATTATTTGGGGAATGA | 52 |
|  |  |  | Reverse | TGGAGATATAGAATTCACACAACATAAA |  |
| 38 | RALY | rs6059655 | Forward | GTGAGGAAATCGAGGCTCAG | 58 |
|  |  |  | Reverse | AGGAGAAAGCTGCAGATCCA |  |
| 39 | OCA2 | rs12441727 | Forward | GGGAAGAGACAGCTCCATGT | 60 |
|  |  |  | Reverse | ACAATCCTGGGAGGTACACG |  |
| 40 | MC1R | rs3212355 | Forward | GAGTGAACCCAGGAAGATGC | 59 |
|  |  |  | Reverse | CATCAAAGGCAGACCTCTCG |  |
| 41 | DEF8 | rs8051733 | Forward | AGGCGGTGGTCTCTCTCTC | 60 |
|  |  |  | Reverse | TTGCAACAGGAGGGTCTAGG |  |
